# Rapid and temporary improvement of depression and anxiety observed following niraparib administration: a case report

**DOI:** 10.1186/s12888-020-02590-4

**Published:** 2020-04-15

**Authors:** Benjamin E. Jewett, Merry N. Miller, Libby A. Ligon, Zachary Carter, Ibrahim Mohammad, Gregory A. Ordway

**Affiliations:** 1 Department of Biomedical Sciences, PO Box 70577, Johnson City, 37614 USA; 2 Department of Psychiatry and Behavioral Sciences, East Tennessee State University, 187 Maple Avenue, Johnson City, TN 37684 USA

**Keywords:** Depression, Anxiety, Niraparib, Ovarian cancer, PARP inhibitor, Case report

## Abstract

**Background:**

Cancer patients are disproportionately affected by generalized anxiety and major depression. For many, current treatments for these conditions are ineffective. In this case report, we present a serendipitous case of anxiety and depression improvement following administration of the poly (ADP-ribose) polymerase (PARP) inhibitor niraparib.

**Case presentation:**

A 61-year old woman with a 20-year history of mild depression developed recurrent ovarian carcinoma and was placed on niraparib for maintenance chemotherapy. With the original onset of ovarian cancer, she experienced an episode of major depression that was resolved with sertraline. After recurrence of ovarian cancer, she experienced a recurrence of major depression and a new onset of generalized anxiety that failed to completely respond to multiple medications. After beginning niraparib therapy the patient noticed a rapid resolution of the symptoms of her anxiety and depression, an effect that was limited to 10–14 days. Due to bone marrow suppression, the patient was taken off and restarted on niraparib several times. Each discontinuation of niraparib resulted in return of her depression and anxiety, while each recontinuation of niraparib resulted in an improvement in her mood and anxiety.

**Conclusions:**

This case demonstrates rapid and temporary improvement of anxiety and depression following niraparib administration. There is ample preclinical data that PARP signaling may play a role in psychiatric illness. A small amount of indirect data from clinical trials also shows that niraparib may have psychiatric benefits. Further research on PARP inhibition and its potential psychoactive effects is sorely needed.

## Background

Patients suffering from cancer are particularly vulnerable to depression and anxiety, having a rate of major depressive disorder and generalized anxiety disorder several times higher than that of the general population [1, 2]. Cancer patients with comorbid major depressive disorder (MDD) have higher mortality rates than those who do not, and there is evidence that the chronic stress state associated with MDD may accelerate tumor growth and invasiveness through a variety of mechanisms [3]. Unfortunately, approximately 30% of patients with depression and anxiety do not respond or have only a partial response to antidepressant treatment [4], further raising risk of poor outcomes in cancer patients.

In this paper, we present a case of rapid temporary remission of depression and generalized anxiety disorder in an ovarian cancer patient treated with niraparib, a small molecule inhibitor of poly (ADP-ribose) polymerase (PARP). This case, along with a large number of studies demonstrating neuroprotective effects of PARP inhibitors, indicates that further investigation of potential psychotherapeutic effects of PARP inhibitors is warranted.

## Case presentation

A 61-year old woman presented to her primary care doctor complaining of chest pain. She had a past medical history of hypothyroidism and 6 years earlier was diagnosed with stage IIIC papillary carcinoma of the right ovary, for which she underwent surgery and chemotherapy. Imaging and laboratory testing confirmed recurrence of ovarian carcinoma, and she again underwent surgery and was placed on chemotherapy with paclitaxel and carboplatin. There was a good therapeutic response to this therapy; her CA-125 levels normalized, and she became clinically asymptomatic.

Prior to her cancer, the patient had a long history of depressive symptoms treated with a variety of antidepressants. She reported mild depressive symptoms associated with recurrent headaches following a hysterectomy at age 35. She had been treated with several antidepressants with partial benefit, including nortriptyline, citalopram, paroxetine, trazodone, and venlafaxine. It is noted that the patient had a family history of depression and anxiety in her mother, who did not respond well to treatment with antidepressants. The patient was first diagnosed with a major depressive episode after her initial cancer diagnosis, which was followed by the deaths of both of her parents. She was treated with sertraline for several years with benefit, but had further headaches and discontinued the sertraline for 4 years. At the time of her cancer recurrence, she experienced an acute worsening of her depression and the new onset of severe anxiety. Additional stressors included her concerns about illnesses that her grandson and best friend were experiencing. At this time (after cancer recurrence), she was diagnosed by her psychiatrist with recurrent MDD and generalized anxiety disorder. She was treated with escitalopram 20 mg/day, augmented with buspirone 30 mg/day (4 months after starting escitalopram), as well as lorazepam 0.25 mg as needed for anxiety. Despite these therapies, she continued to experience significant depression and anxiety.

Nine months after her cancer recurrence and following normalization of her CA-125 levels, and 6 months after starting escitalopram, the patient was started on maintenance oral chemotherapy with niraparib 300 mg at bedtime. The patient reported a dramatic response in her mood after initiating treatment with niraparib, and she awoke the next morning feeling no depression or anxiety. She was very surprised to feel so good, as she had not experienced a day free of depression and anxiety since her cancer recurrence. This mood improvement persisted for approximately 2 weeks.

Unfortunately, the patient developed side effects to niraparib, including insomnia, thrombocytopenia, and neutropenia. One month after initiation, niraparib was held for 2 weeks due to the thrombocytopenia. During this 2 week period, the patient described a worsening of her mood, particularly her anxiety. Niraparib was then restarted at a decreased dose of 200 mg/day. The patient again experienced a dramatic improvement in mood and anxiety—an effect that lasted for 10 days.

Niraparib was temporarily held again 2 months later due to neutropenia, and was later restarted at the dose of 100 mg/day. Again the patient described experiencing a large improvement in her depression and especially in her anxiety when the niraparib was restarted. Again on this occasion, the improvement lasted about 10 days. Eventually, due to thrombocytopenia and neutropenia, niraparib dosing was lowered to 100 mg every other day.

The patient’s psychiatric medication regimen was adjusted during the same time period she was being treated with niraparib. While on niraparib, the patient was able to decrease her lorazepam usage down from 0.25–0.50 mg per day to 0.25–0.50 mg once a week. Several weeks after the niraparib was begun, the patient was weaned off escitalopram and buspirone due to lack of efficacy, and she was begun on desvenlafaxine 50 mg/day and mirtazapine 45 mg/day. Five months later, she was weaned off mirtazapine but stayed on desvenlafaxine 50 mg. The patient reported only partial benefit from these antidepressant medications, in contrast to the complete, albeit temporary, symptomatic resolution she initially experienced with niraparib.

For this report, the patient was interviewed by board-certified psychiatrist coauthor MNM approximately 10 months after her initial treatment with niraparib. MNM conducted a psychiatric interview and administered three self-report evaluations (Patient Health Questionnaire, PHQ-9; Generalized Anxiety Disorder-7 item, GAD7; Mood Disorder Questionnaire, MDQ). At the time of the interview, the patient’s cancer continued to be in remission. As a result of the interview and self-assessments, it was determined that her mood continued to be mildly dysphoric and anxious but was overall stable on 50 mg desvenlafaxine daily and 100 mg of niraparib every other day. There was no evidence of hypomania or mania in the MDQ, and also no evidence of bipolar disorder in the patient’s history.

## Discussion and conclusions

There are several interesting features of this patient’s response to niraparib that should be noted. First, the onset of the mood improvement following initiation of niraparib therapy was rapid, taking effect within 12 h. Likewise, upon discontinuation of niraparib, anxiety and depression symptoms rapidly returned. Second, it is important to note that the patient’s symptomatic response to niraparib appeared time-limited, with symptom resolution lasting only 10–14 days. Third, there did not appear to be a clinical difference in mood improvement following doses of 300 mg versus 100 mg of niraparib, while the dose decrease did reduce the rate of side effects.

The mechanism of niraparib’s potential psychoactive effects is currently unclear. PARP inhibitors are FDA-approved for the treatment of BRCA-positive breast cancer and ovarian cancer [5]. PARPs are a family of enzymes that perform post-translational modification to proteins to facilitate cellular processes, including DNA base excision repair. PARPs, particularly PARP1 and PARP2, are commonly considered DNA base excision repair enzymes. However, activation of PARP1 is also directly linked to inflammation cascades, including activation of NF-κB and production of downstream cytokines. Besides anti-cancer effects, PARP inhibitors have anti-inflammatory and neuroprotective properties and have demonstrated predicted therapeutic activity in animal models of diseases that involve oxidative stress and inflammation, including Parkinson’s disease [6], Alzheimer’s disease [7], ischemic brain injury [8], and most recently depression [9]. PARP may lie in a pathway leading to neuroinflammatory cascades in MDD. In fact, elevated PARP1 gene expression has been reported in postmortem brain tissue from MDD donors [10] and from blood cells from patients with MDD [11]. The rapidity of the effect of niraparib on mood in the present case might seem to preclude the possibility that effects on mood were secondary to an anti-inflammatory action. However, PARP activation modulates the expression of IL-1β and other cytokines [12] that have been shown to have direct actions on brain monoamines, including dopamine, norepinephrine, and serotonin [13–15]. The temporary nature of the mood benefit possibly elicited by niraparib in this patient is also noteworthy. Further research on the mechanisms by which PARP inhibitors affect mood is sorely needed.

There are two drugs with PARP inhibitory activity that have antidepressant or mood stabilizing properties, although a connection between effects on PARP and mood have not been previously postulated. Minocycline is a potent PARP inhibitor [16] that also has robust antidepressant effects [17]. Laboratory studies have also suggested that lithium may modulate PARP signaling [18]. Recently, two PARP inhibitors, 3-aminobenzaminde and 5-isoquinolinone, have been shown to demonstrate antidepressant-like activity in preclinical rodent models (social defeat and chronic unpredictable stress, forced swim test) with efficacy similar to fluoxetine. Additionally, PARP inhibition appeared to have an additive antidepressant effect when combined with fluoxetine [9]. PARP inhibitors have also been shown to protect rodents from helplessness behavior in inflammation-induced sickness behavior [19].

We examined data from clinical trials of PARP inhibitors reported at clinicaltrials.gov to determine whether there was prior evidence of mood improvement in cancer patients. Only placebo-controlled trials with group sample sizes of > 20 were considered. It should be noted that cancer drug trials are not designed to evaluate mood effects and do not use robust psychiatric rating scales. Several clinical trials of PARP inhibitors report little or no anxiety and (or depression when measured) in enrolled cancer patients. Two trials of veliparib that reported anxiety in some placebo-treated patients showed a modestly lower incidence of anxiety in PARP inhibitor-treated patients [20, 21]. In contrast, no such trend was observed for placebo-controlled trials of oliparib. No results from clinical trials using niraparib were posted at the time of writing of this report.

There are many factors that limit the conclusions that can be drawn from this case report. First, the clinical course of only one patient is described. This patient was medically complicated and was treated with multiple psychoactive drugs during the course of the period covered by this report. While there is a temporal relationship between the patient’s symptomatic relief and initiation of niraparib, there is no way to definitely state that niraparib resolved the patient’s depression and anxiety. Also, each time the niraparib was begun, her improvement in mood was temporary with the recurrence of some of her symptoms after 10 days to 2 weeks. Brain penetration and retention for many PARP inhibitors is poor and is a deterrent to treating brain cancers [22]. In contrast, niraparib crosses the blood brain barrier and accumulates in brain tissue [23]. Finally, niraparib likely contributed to the development of thrombocytopenia and neutropenia in this patient.

To our knowledge, this is the first published report of putative mood effects for a PARP inhibitor in a human subject. Of course, this is not the first case of a non-psychiatric drug demonstrating positive psychiatric effects. Chlorpromazine, the first antipsychotic, was initially used as a component of general anesthesia, and its psychoactive effects were first noted by a surgeon [24]. Likewise, iproniazid, the first monoamine oxidase inhibitor, was initially developed as an anti-tubercular agent and its psychoactive effects were only fortuitously discovered later [25].

While patients often bring negative psychiatric side effects to the attention of clinicians, positive psychotropic effects of drugs may often be overlooked. This case report underlines the need for more research on the relationship between PARP signaling, depression, and anxiety at the preclinical, translational, and clinical levels.

## Abbreviations

MDD: Major depressive disorder
PARP: Poly (ADP-ribose) polymerase
FDA: U.S. Food and Drug Administration
CA-125: Cancer antigen 125 protein
BRCA: Breast cancer genes
NF-κB: Nuclear factor kappa-light-chain-enhancer of activated B cells
IL-1β: Interleukin 1 beta
